# Prevalence and risk factors of poor immune recovery among adult HIV patients attending care and treatment centre in northwestern Tanzania following the use of highly active antiretroviral therapy: a retrospective study

**DOI:** 10.1186/s13104-017-2521-0

**Published:** 2017-06-08

**Authors:** Daniel W. Gunda, Semvua B. Kilonzo, Erasmus Kamugisha, Engelbert Z. Rauya, Bonaventura C. Mpondo

**Affiliations:** 1 Department of Medicine, Weill Bugando School of Medicine, 1464 Mwanza, Tanzania; 2 Department of Biochemistry and Molecular Biology, Weill Bugando School of Medicine, 1464 Mwanza, Tanzania; 3 Care and Treatment Center, Sekouture Regional Referral Hospital, 132, Mwanza, Tanzania; 4 Department of Medicine, University of Dodoma, School of Medicine, 395, Dodoma, Tanzania

**Keywords:** HIV, Immune recovery, Antiretroviral therapy, Northwestern Tanzania

## Abstract

**Background:**

Highly Active Antiretroviral therapy (HAART) reverses the effect of Human Immunodeficiency Virus/Acquired Immune Deficiency Syndrome (HIV/AIDS) by durably suppressing viral replication. This allows CD4 gain to levels that are adequate enough to restore the body’s capability to fight against opportunistic infections (OIs). Patients with poor immune recovery have been shown to have higher risk of developing both AIDS and non AIDS related clinical events. This study aimed at assessing the proportions and risk factors of poor immune recovery in adult HIV-infected patients on 48 months of HAART attending care and treatment center (CTC) in northwestern Tanzania.

**Methods:**

A retrospective analysis of adult HIV patients’ data attending CTC at Sekou Toure hospital and who initiated HAART between February 2004 and January 2008 was done. Poor immune recovery was defined as a CD4 count less than 350 cells/µl on follow up as used in other studies.

**Results:**

A total of 734 patients were included in the study. In this study 50.25% of patients attending CTC at Sekou Toure hospital were found to have poor immune recovery. The risk of developing inadequate immune recovery was independently associated with male gender, age older than 50 years, low baseline CD4 counts, and advanced World Health Organization (WHO) clinical stage.

**Conclusions:**

Poor immune recovery is prevalent among adult HIV patients attending CTC at Sekou Toure hospital in Northwestern part of Tanzania and opportunistic infections are common in this sub group of patients. Clinicians in resource limited countries need to identify these patients timely and plan them for targeted viral assessment and close clinical follow up to improve their long term clinical outcome.

## Background

HIV is known to infect CD4 positive cells and its replication in these immune cells causes progressive lysis and reduction of the number and quality of functional immune cells [1–3]. With time the body fails to contain the viral replication and immune deficiency sets in, being marked by low CD4 counts with increased morbidity and mortality from opportunistic infections [4]. The use of potent HAART is intended to induce sustained suppression of HIV viral replication allowing CD4 regain and restore the body’s ability to fight against opportunistic infections [5–9]. Since the arrival of HAART it has been shown that there is a significant reduction of both AIDS and non AIDS related morbidity and mortality in People Living With HIV/AIDS (PLWHA) [5, 10–14], especially in those who achieve an adequate immune recovery. In most patients adequate CD4 regain is characterized by an increase of about 50-150 cells/µl in the first year followed by 50–100 cells/µl in subsequent years [15]; reaching an immunological threshold of CD4 >350–500 cells/µl in 4–7 years of effective HAART [8, 16, 17]. This immunological threshold is clinically important in view of the fact that patients who regain their CD4 to this immunological point have a better clinical outcome with non AIDS events almost comparable to normal populations [18–23]. Otherwise patients whose rate of CD4 regain is less than 50 cells/µl a year may possibly take a much longer time [24–27], before reaching this immunological threshold and this subgroup of patients has been shown to be at higher risk of both AIDS and non AIDS related morbidities and mortalities [28–31]. Poor immune recovery represents a very frequent problem in clinical practice worldwide with a prevalence which can be in excess of 50%. In view of this information WHO recommends early identification of patients who fail on HAART by using clinical-immune monitoring as a surrogate marker of treatment response and plan them for targeted virological confirmation. Even though poor immune response is so prevalent, there is no prior documentation of the magnitude of this problem in Tanzania. Our study aimed at determining the proportion and risk factors of poor immune recovery in adult HIV patients on standard HAART for 48 months in Northwestern Tanzania. This will assist clinicians in Tanzania and other resource limited settings to be able to identify potential patients who may possibly benefit from targeted viral load testing and close clinical follow up.

## Methods

This was a retrospective study involving all adult HIV positive patients (age ≥18 years) who started on antiretroviral therapy at Sekouture hospital care and treatment center. Children and adult patients who were yet to be on ART were excluded. Sekouture is a regional referral hospital located in Mwanza, in north western part of Tanzania. A retrospective analysis of 4 years data of HIV patients initiated on HAART between 20th February 2004 and 20th January 2008 was done. In this study HAART was referred to as a combination of 2 Nucleoside reverse transcriptase and one non nucleoside reverse transcriptase inhibitor or a protease inhibitor with or without a pharmacological booster.

The variables collected included age, sex, marital status, occupation, baseline CD4 count, occurrence of opportunistic infections within 4 years of follow up at CTC, body weight and CD4 count at 48 months of follow up. Data were managed using Epi Data 3.1 (CDC Atlanta, US) and analysis was done using STATA version 12 (College Station, Texas, US). The baseline CD4 counts were categorized into two using cut off point CD4 of 200 cells/µl and patients with CD4 counts of less than 200 cells/µl at base line were referred as presenting with severe immune suppression. The immune response at 48 months of follow up was also divided into two groups with cut off point CD4 of 350 cells/µl as used in other studies [32]. Immune reconstitution was defined as CD4 count ≥350 cells/µl; at 48 months of follow up and those patients who had CD4 counts <350 cells/µl at 48 months were defined as having poor immune recovery. The categorical variables were summarized as proportions and their significance of difference in distribution within the categories of immune response was assessed using Pearson’s Chi square test or Fisher’s exact test where appropriate. Parametric continuous data were summarized as mean with standard deviation and the significance of difference in means within the two groups was assessed using one way analysis of variance (ANOVA). Non-parametric continuous data were summarized as median with interquartile range and the difference in medians within the categories of immune response was compared using Kruskal–Wallis equality-of-populations rank test. The odds ratios (OR) and 95% confidence intervals (CI) of risk factors associated with poor immune recovery were calculated using univariate logistic regression model followed by multivariate logistic regression model. All risk factors associated with poor immune recovery in the univariate analysis with *p* values less than 0.05 were considered for inclusion in the multivariate model. A stepwise approach was used to derive a Parsimonious model, and all associated factors in the final model were considered significant if the *p* value was less than 0.05.

## Study results

In total 734 patients were included in this study; in which most patients were females (66.76%), with a median age of 39.03(21–60) years. Most of patients (52%) were married, unemployed (89%) and in WHO clinical stage 3 and 4 (57.49%). Of these patients 722 (98.4%) had recorded baseline CD4, where more than 75 percent; 545 (75.48%) had baseline CD4 counts of less than 200 cells/µl, while 670 (92.27%) had baseline CD4 counts <350 cells/µl as summarized in Table 1. And at 48 moths of follow up, 593(80.8%) of 734 patients had recorded CD4 counts; of these 289 (50.25%) had CD4 count <350 cells/µl. Patients with poor immune recovery were more likely to be male in gender (OR = 1.52, p = 0.039) and older than 50 years (OR = 4.17, p < 0.001), with baseline CD4 count of less than 200 cells/µl (OR = 4.32, p < 0.001), in WHO clinical stage 3 and 4 (OR = 1.47, p = 0.043). Patients with poor immunological recovery also had higher occurrence of an opportunistic infection (OR = 4.32, p < 0.001). The difference in distribution of other factors was not significant including the type of opportunistic infections as summarized in Table 2.

**Table 1:** The Baseline demographic, clinical and laboratory characteristics of the study participants

Factor	Frequency of observation	Percentage/mean (IQR)
Gender		
Male	244	33.24
Female	490	66.76
Age (years)	734	39.03 [21–60]
Marital status		
Single	115	15.67
Married	388	52.86
Divorced	96	13.08
Widowed	135	18.39
Occupation		
Employed	656	89.37
Unemployed	78	10.63
Enrolment WHO stage		
Stage 1 and 2	312	42.51
Stage 3 and 4	422	57.49
BMI index (kg/m^2^)		
Underweight(<18.5)	22	29.33
Normal (18.5–24.9)	49	65.33
Overweight(25–29.9)	04	05.33
Obese(>30.00)	00	00.00
Baseline cd4(cells/µl)	722	147.2[1–664]
Baseline CD4 levels		
<200 cells/µl	545	75.48
>200 cells/µl	177	24.52
<350 cells/µl	670	92.27
Missing data	012	01.63

*MO* months, *CD4* cluster of differentiation 4, *BMI* Body Mass Index, *WHO* World Health Organization, *IQR* inter quartile range

**Table 2:** The univariate and multivariate analysis of risk factors associated with poor immune recovery following the use of HAART for 48 months

Predictive factor	CD4 recovery at 48 months		Unadjusted		Adjusted	
	Inadequate recovery (CD4 < 350)	Adequate recovery (CD4 > 350)	OR (95% CI)	P value	OR(95% CI)	P value
Gender						
Male	117 (39.26)	083 (28.14)				
Female	181 (60.74)	212 (71.86)	1.65 (1.17–2.33)	0.004	1.52 (1.02–2.27)	0.039
Age (years)						
>50	227 (76.17)	274 (92.88)				
≤50	071 (23.83)	021 (07.12)	4.1 (2.43–6.84)	0.000	4.17 (2.31–50)	0.000
Occupation						
Employed	032 (10.74)	034 (11.53)				
Unemployed	266 (89.26)	261 (88.47)	–	–	–	–
WHO stage						
Stage 1 and 2	105 (35.23)	139 (47.12)				
Stage 3 and 4	193 (64.77)	156 (52.88)	1.64 (1.18–2.28)	0.003	1.47 (1.01–2.14)	0.043
Baseline CD4						
<200 cells/µl	260 (88.44)	187 (64.71)				
≥200 cells/µl	034 (11.56)	102 (35.29)	4.17 (2.71–6.42)	0.000	4.32 (2.70–6.90)	0.000
Baseline BMI						
Underweight	10 (31.25)	09 (27.27)				
Normalweight	22 (68.75)	20 (60.61)				
Overweight	–	04 (12.12)	0.92 (0.78–1.10)	0.332		
Obese	264 (88.59)	–				
Opportunistic INF						
Yes	212 (71.14)	118 (40.00)				
No	086 (28.86)	177 (60.00)	3.69 (2.63–5.20)	0.000	3.91 (2.69–5.69)	0.000
Type of OI						
TB	169 (79.72)	104 (85.95)				
Others (D, C)	043 (20.28)	017 (14.05)	1.56 (0.84–2.87)	0.157		

*D* diarrhea, *C* candidiasis, *TB* tuberculosis, *BMI* Body Mass Index, *WHO* World Health Organization, *INF* infection, *OI* opportunistic infection, *OR* odds ratio

## Discussion

The aim of this study was to determine the proportion and risk factors of poor immunological response to HAART in adult HIV patients attending CTC in northwestern Tanzania for 48 months. Of the 593 patients who had records of their CD4, 289 (50.25%) did not reach an immunological threshold of ≥350 cells/µl at 4 years of HAART. This prevalence seems slightly higher than those reported from developed countries. In one study in Australia Kaufmann et al. indicated that about 14% of the virologically suppressed patients didn’t reach an immunological cut off point of 350 cells/µl at four years of HAART [8]. Another study in United States of America (USA) defining inadequate immune recovery as a CD4 gain of less than 150 cells/µl a year reported a prevalence of 36% in patients who were virologically suppressed [33]. While a study in San Francisco with CD4 count cut off of 350 cells/µl; 41% did not reach this immunological threshold [16]. Of note from these studies as might be true with most of other studies from developed countries these rates represent poor immune recovery in patients who were virologically suppressed. Our study represents one of the studies from resource limited settings where virological monitoring of treatment response is not obtainable and feasible for routine practice. In these settings patients with inadequate immune recovery is a heterogeneous group which takes in both concordant and discordant non responders making the prevalence higher than what is reported from studies that exclude concordant non responders [34]. In Uganda; Nanzigu et al. reported a slightly higher rate of poor immune recovery (62.9%) at 5 years with cut off point of 418 cells/µl [35]. However; even with these differences, the clinical relevance drawn from these studies remains similar. Previous studies have indicated that patients with poor immune response to HAART have increased risk of AIDS progression with higher rates of both AIDS and non AIDS related clinical events [12, 29, 36], even with complete virological suppression. Similarly in our study we observed that patients with poor immune recovery had higher rates of OI as compared to those who reached the immunological threshold of >350 cells/µl (71.14% vs. 40%, p < 0.001). In our study we found that male gender was strongly associated with poor immune recovery where male patients were 1.52 times more likely to have poor immune recovery than females. Several other studies have indicated that female patients have a better and faster immune recovery than men [16, 26, 35, 37]. This may possibly be explained by the hormonal regulatory effect on CD4 numbers. In this regard females have been shown to have comparatively higher CD4 level than men which is estrogen regulated [38], and with use of suppressive HAART this normal homeostatic regulation of CD4 cell number may therefore lead into higher CD4 gain in females than men [37]. The other risk factor was being older than 50 years (OR = 4.17, p < 0.001). This is in agreement with other studies [35, 36, 39]. Older patients probably have reduced thymus functions and other degenerative processes as suggested in most studies [8, 36, 40–42]. Clinically this suggests that there should perhaps be screening programs in ages close to 50 for early diagnosis and timely initiation of HAART. A baseline CD4 count <200 cells/µl was another risk factor which was independently associated with inadequate immune recovery, (OR = 4.32, p = 0.001). Low CD4 counts represent a severe immune destruction and patients who start ART at lower baseline CD4 bellow 200 cells/µl have been demonstrated to have a poor immune recovery [24, 36, 43]. Hunt et al. demonstrated that only about 21–49% of patients who start ART at CD4 counts of <200 cells/µl achieved CD4 counts of 350–500 cells/µl at 4 years while more than 93% of those who initiated HAART at CD4 levels >200 to 350 cells/µl attained CD4 levels >350 to 500 cells/µl at four years [16]. This emphasizes further on the value of starting HAART at cut off point of CD4 ≥350 cells/µl [13, 44]. It was also observed that patients in WHO stage 3 and 4 were 1.47 times more likely to have inadequate immune recovery on receipt of HAART as compared to those in WHO stage 1 and 2 (OR = 1.47, p = 0.043). Kaufmann et al. had similar observation that patients in advanced HIV stage had poor immune recovery as compared to those in early stage of the disease [8]. These findings suggest that patients who present late in advanced HIV/AIDS and severe immune suppression need a much closer clinical follow up to improve their outcome.

This study had a number of limitations. It was a single site clinic based study and therefore findings may not be generalizable to the general population. Being a retrospective study, the data was obtained from patients’ files; these are most of times incomplete; some of the important variables were missing including information on compliance to medications and missing CD4 counts at 48 months. In this study about 141(19.21%) patients had missing CD4 records at 48 month, on a sub analysis (not shown) most of them 98(70.5%) had baseline CD4 <200 cells/µl indicating that they were more likely to have poor immune response at 48 months of follow up and this could have made the burden of poor immune recovery probably more worse. However even with these limitations our study has a number of strengths inclusive of being a relatively large sample size study that describes immune recovery in patients initiated on HAART at low baseline CD4 counts.

## Conclusions

Poor immune recovery is common in Northwestern Tanzania. This is strongly associated with male gender, older age than 50 years, low baseline CD4 count, advanced clinical stage and occurrence of opportunistic conditions. Clinicians from resource limited settings should identify potential patients and plan them for a much closer clinical follow up and targeted plasma viral load since this subgroup of patients have poor clinical outcomes.

## Abbreviations

AIDS: acquired immune deficiency syndrome
ANOVA: analysis of variance
BMC: Bugando medical center
BMI: Body Mass Index
CD4: cluster of differentiation 4
CDC: Center for Disease Control
CTC: care and treatment Center
CUHAS: Catholic University of Health and Allied Sciences
HAART: highly active antiretroviral therapy
IQR: inter quartile range
OIs: opportunistic infections
OR: odds ratio
PLWHA: people living with HIV/AIDS
US: United States
USA: United States of America
WHO: World Health Organization
